# Carotid Dissection and Isolated Paralysis of Ipsilateral Half Tongue: Clinical Cases

**DOI:** 10.1155/2020/8853206

**Published:** 2020-12-17

**Authors:** Komi Igneza Agbotsou, Damelan Kombate, Christopher Mehri, Kossivi Apetse, Kpalma Duga Bakpatina-Batako, Olivier Guerrier, Albert Beschet, Karine Blanc Lasserre, Ludovic Breynaert, Victor Chan

**Affiliations:** ^1^Neurology Department of Valence Hospital Center, Valence, France; ^2^Neurology Department of Campus Teaching Hospital, Lome, Togo

## Abstract

**Objective:**

We report two cases of carotid dissection revealed by isolated paralysis of the ipsilateral half tongue. *Observations*. First patient, 52 years old, with no particular medical or surgical history, presented with isolated paralysis of the left half tongue preceded by two weeks of moderate-intensity cervicalgia and having been the subject to cervical manipulation. MRI revealed dissection of the left internal carotid artery in its prepetrous portion. The evolution after 6 weeks of platelet aggregating inhibitors treatment was favorable. The second patient, 74 years old, with no particular medical or chirurgical history, presented with a sudden onset of paralysis of the left half tongue preceded by unusual headaches associated with neck pain. Brain MRI showed aneurysmal ectasia of the left internal carotid with parietal irregularity suggestive of carotid dissection. The evolution after four weeks of treatment with anticoagulant was favorable.

**Conclusion:**

Carotid dissection revealed by isolated paralysis of the half tongue is rare. It is generally of good prognosis. However, in paralysis of half tongue, it must be urgently sought and treated to reduce the risk of a transient or constituted ischemic accident.

## 1. Introduction

Arterial dissection is defined by the formation of a hematoma within the lining of the artery, communicating or not with arterial light through a breach in the intima, cleaving the parietal leaflets. The result is an occlusion, stenosis, or aneurysmal dilation of the vessel [1]. A dissection of the internal carotid artery causes paralysis of the large hypoglossal nerve either by a compressive mechanism via the wall hematoma on the nerve or by a mechanism of vascular interruption of the vasa nervorum feeding the nerve [2].

Internal carotid artery dissections (ICA) are the most common dissections of the cervical end-brain arteries with cerebral infarction with an estimated incidence of 3/100,000 cases per year, affecting both sexes with an average age of 45 years [3, 4].

Carotid dissection may be responsible for the damage to the lower cranial nerves including the glossopharyngeal nerve (IX), the vagus nerve (X), the spinal nerve (XI), and the large hypoglossal nerve (XII). This is a very rare situation found in 8% of cases [5]. The damage to the hypoglossal nerve is most commonly found and is observed in 5% of patients [5].

Some cases of carotid dissection revealed by isolated paralysis of the half tongue have been described in the literature. We therefore set ourselves the goal of reporting two cases of carotid dissection revealed by isolated paralysis of the ipsilateral half tongue and literature review.

## 2. Patients and Methods

This was a retrospective and descriptive study of two cases of carotid dissection revealed by isolated paralysis of the ipsilateral half tongue. The study involved two patients, the first aged 52 and the second aged 74, who were admitted to the neurovascular unit of the neurology department of the Valencia Hospital Center. The diagnosis of carotid dissection was evoked before clinical signs suggestive of isolated paralysis of the large hypoglossal nerve. Diagnostic confirmation was performed by magnetic resonance imaging (MRI) of the neck vessels. Sociodemographic, clinical, radiological, therapeutic, and evolutionary data were studied.

## 3. Cases Presentation

### 3.1. Observation No. 1

Mr. L.D. born on July 20, 1965, a trader, right-handed, self-employed, with no cardiovascular risk factor or a personal or family pathological history, was admitted on December 30, 2017 for difficulties in moving his tongue. On December 29, 2017, he presented suddenly difficulties in mobilizing his tongue, at lunchtime with some difficulties in carrying the food bowl and chewing associated with articulatory difficulties. These symptoms were preceded 48 hours by left, unusual, and mild hemicranial headaches, partially relieved by paracetamol associated with cervicalgia and no vomiting. There was no infectious episode or traumatic context.

**Figure 1:**
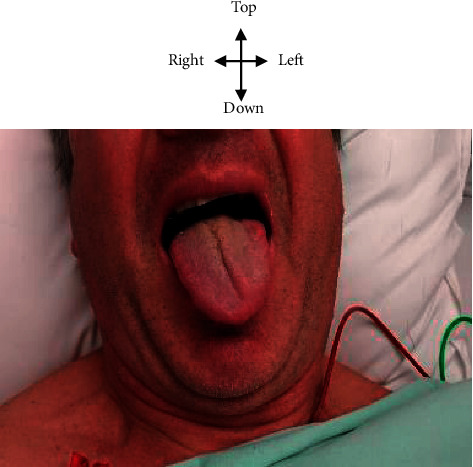
Left ipsilateral deviation of the tongue to protraction in Mr. L.D. in favor of a left carotid dissection.

Mr. L.D.'s physical examination recorded normal hemodynamic constants with temperature at 37 degrees Celsius, blood pressure at 120/70 mmHg, pulse at 70 pulses per minute, and weight at 72 kilograms for a height of 175 cm (BMI at 23.51 kg/m^2^). The general condition was good. The neurological examination had found a deviation of the tongue to the left when he pulled the tongue and vice versa when he returned it, a left lingual hemiatrophy. The consciousness was normal. The physical examination did not find any change in the taste or impairment of the other cranial pairs. Mr. L.D did not have a sensory deficit, nor a motor deficit with the limbs or the face. There was no osteotendon reflex disorder or Babinski sign. Cerebellar, proprioceptive, or vestibular ataxia was not found. The neck was flexible without the sign of Kernig or Brudzinski. The standing position with joined feet was correct when opening or closing eyes. The rest of the examination including cardiovascular, pleuropulmonary, and digestive was normal.  Figure 1 shows Mr. L.D exhibiting an ipsilateral left deviation of the tongue when protracting, suggestive of a left carotid dissection.

**Figure 2:**
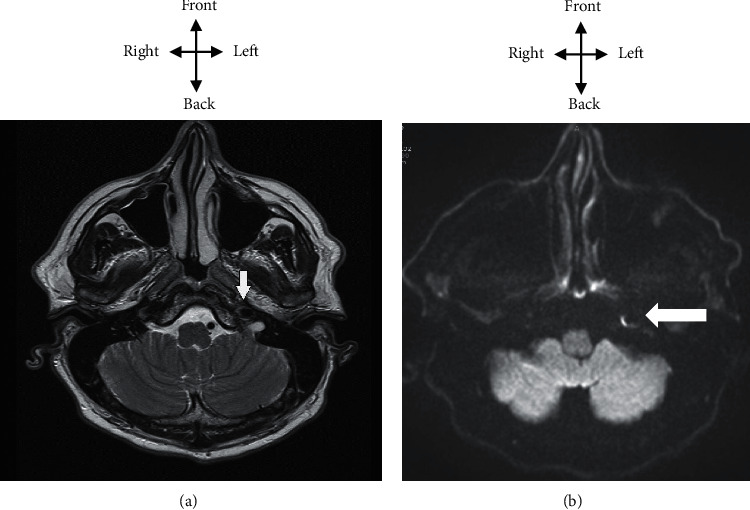
MRI of the neck vessels, T2 TSE axial (a) and diffusion axial (b), aneurysm ectasia of the left internal carotid with parietal irregularity.

Brain MRI (broadcast sequences, FLAIR, and ADC mapping) was normal. MRI of the neck vessels confirmed an aspect of wall hematoma in the left internal carotid artery in diffusion compatible with a dissection of the left internal carotid artery as shown in Figure 2. *C*-reactive protein was at 4 mg/l. At the blood formula count, the leukocyte level was 4500 cells/ml. Creatininemia was at 9 mg/l with a clearance at 105 ml/min. Retroviral serology was negative, as hepatitis and syphilis serologies. Total cholesterol was at 1.50 g/l, triglycerides at 1.10 g/l, HDL cholesterol at 0.50 g/l, and LDL cholesterol at 0.68 g/l. The autoimmune assessment was negative.

M. L.D. received treatment with a platelet aggregating inhibitor (acetyl salicylic acid 160 mg per day at meals) according to the cervical artery dissection in stroke study (CADISS), a level 1 analgesic treatment (paracetamol compressed one gram every 6 hours for headaches and cervicalgia) and speech therapy.

The evolution was favorable marked by regression of headache, cervicalgia, and paralysis of the half tongue after six weeks.

### 3.2. Observation No. 2

Mr. C.E. born on September 28, 1942, right-handed, retired gendarme, married, autonomous, without cardiovascular risk factor, without a personal or familial pathological history, transferred by the emergency department of the Valencia Hospital to the neurology department on May 26, 2016, for the management of an isolated paralysis of the half tongue in a context of cervicalgia.

Fifteen days before his admission to the Valencia Hospital Center, he presented moderate-intensity mild neck pains that appeared without triggering factors or infectious contexts and that has increased on May 23, 2016. He then consulted his physiotherapist the next day who performed cervical manipulations on suspicion of cervical osteoarthritis. On May 25, 2016, he noticed a solid swallowing disorder. He consulted his physician who addressed him to the emergency department on May 26, 2016, where he was transferred to the neurology department.

**Figure 3:**
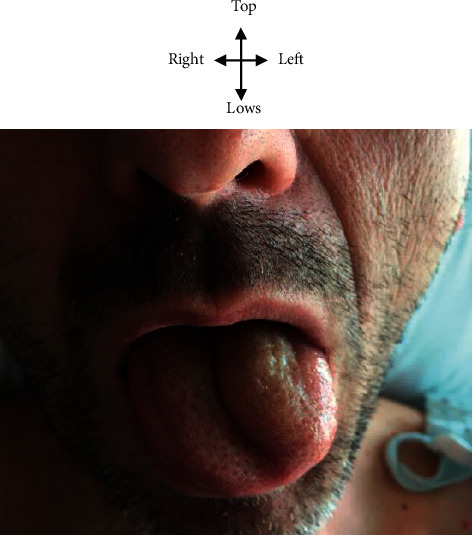
Left ipsilateral deviation of the tongue to protraction (arrow) in Mr. C.E. suggestive of a left carotid dissection.

**Figure 4:**
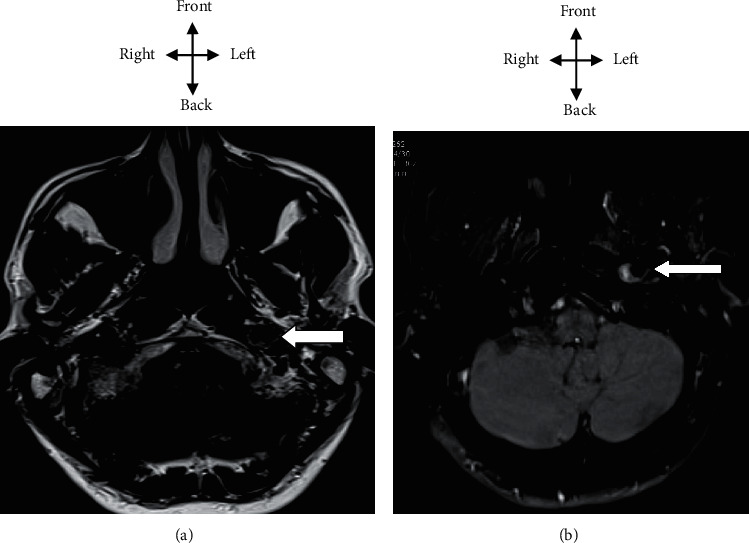
MRI of the neck vessels, T2 FLAIR axial (a) and T1 FAT-SAT axial (b), hyperintense subintimal hematoma.

In the neurology department, the general examination found normal hemodynamic constants with temperature at 37.1° Celsius, blood pressure at 110/60 mmHg, pulse at 75 pulses per minute, and weight at 70 kilograms for a height of 170 cm (BMI at 24.22 kg/m^2^). The general condition was good. The consciousness was normal. Mr. C.E. had a mild neck pain. Examination of the cranial pairs had found paralysis of the left XII nerve with deviation of the tongue to the left when tracting it out and vice versa when it enters it, a left lingual hemiatrophy and swallowing disorder. He had no sagging of the velum, and the nauseous reflex was present. There was no impairment of the other cranial pairs. Mr. C.E. did not have a sensitive or motor deficit at the limb and face. Osteotendon reflexes were present and normal, and the plantar skin reflex was in bilateral flexion. There was no cerebellar, proprioceptive, or vestibular ataxia. The neck was flexible without the sign of Kernig, nor the sign of Brudzinski. The rest of the examination including cardiovascular, pleuropulmonary, and digestive was normal.  Figure 3 shows M.C.E. patient with a tongue ipsilateral deviation of the left suggesting a left carotid dissection. A brain MRI and neck vessels confirmed a subintimal hematoma of the left internal carotid artery in favor of a left carotid dissection as shown in Figure 4.

The *C*-reactive protein was at 4.5 mg/l. At the blood formula count, the leukocyte level was 5100 cells/ml. Creatininemia was 8.5 mg/l with clearance at 102 ml/min. Retroviral serology was negative, as hepatitis and syphilis serologies. Total cholesterol was at 1.40 g/l, triglycerides at 1.05 g/l, HDL cholesterol at 0.55 g/l, and LDL cholesterol at 0.60 g/l. The autoimmune assessment was negative.

The final diagnosis was an extracranial left carotid dissection in Mr. C.E. with no particular medical history with an isolated XII impairment. He benefited from a curative anticoagulation with unfractionate heparin, which was relayed by antivitamin K from day one according to the CADISS (cervical artery dissection in stroke study) with a target between goal of INR between 2 and 3, analgesic treatment level 1 (one gram tablet of paracetamol every 6 hours for headaches and cervicalgia).

The evolution in the service was favorable with a net improvement in cervicalgia after 72 hours. The failure to contract the left tongue associated with swallowing disorders had improved after four weeks without speech therapy. A mixed diet at the beginning and then gradually solid after a week of evolution had been introduced.

## 4. Discussion

Dissections of the cervicoencephalic arteries are responsible for about two percentages (2%) of all brain infarctions and 25% of heart attacks in people under 45 years [6–8]. The annual incidence is in the order of 3 per 100,000 [6]. This rate is underestimated because, based on stroke records, it only takes into account dissections diagnosed with cerebral ischemia and not benign forms, limited to local signs, or the severe forms that cause early death [6]. This incidence of dissections is also underestimated because a dissection may be overlooked because of its asymptomatic, paucisymptomatic (isolated local signs), or even atypical, as in our two patients with isolated paralysis of half tongue, sometimes not prompting a consultation, or a lack of means used to establish the diagnosis. Dissections of the internal carotid arteries (ICA) are the most common with an estimated incidence of 3/100,000 cases per year, affecting both sexes with an average age around 45 years [3, 4]. When the damage to the large hypoglossal nerve by carotid dissection is isolated as in our two patients, it may be unnoticed and delay the diagnosis of carotid dissection [5].

In our first patient, the neurological examination had found a deviation of the tongue to the left when he pulls the tongue and vice versa when he enters it, a left lingual hemiatrophy associated with swallowing disorders, and classic signs of paralysis of the half tongue. This had suggested an isolated paralysis of the half tongue. Our second patient also presented the same clinical picture as the first patient. Nevertheless, careful questioning remains the key to the etiological diagnosis of isolated half tongue paralysis [9]. The history of the disease is essential [9]. We should look for a triggering factor (cervical trauma and recent surgery) in the circumstances of occurence, a favorable background (cardiovascular history, arthropathy, system disease, and malignant tumor), and also associated signs [9]. This helps to guide the paraclinical assessment. However, no revealing context was found in two patients.

Paraclinical explorations of carotid dissection involve an echo-Doppler of neck vessels, a brain and angioscanner scan of the neck vessels, and conventional arteriography with magnetic resonance imaging (MRI) and magnetic resonance angiography (MRA) of the neck vessels [10]. Arterial imaging is a primary part of investigations. Main exam is MRI, with in particular, the T1 fat suppression sequences (FAT-SAT) that allow to notice the wall hematoma, while the angiographic sequences (MRA) show stenosis, occlusion, or dissecting aneurysm and the diffusion sequence that is interesting for high dissection [10]. Sometimes, the hematoma can be visualized using other techniques such as the angioscanner or ultrasound [10, 11]. In both of our patients, MRI and cerebral and neck vessels MRA were performed to show the existence of carotid dissection and eliminated an ischemic stroke whose management differs from that of isolated paralysis of the hall tongue. Other diagnostic radiological check-ups including the echo-Doppler of the supraaortic trunks, the CT scan, and angioscanner of the supraaortic trunks were not carried out in our two patients because of the neurovascular emergency. Indeed, our two patients were admitted to the Valencia Hospital Center in a context of the neurovascular emergencies where the neurovascular emergency check-up to be performed was the brain and neck vessels MRI.

The treatment of isolated paralysis of the half tongue has two objectives: treat the mechanism of the lesion and restore nerve function [9]. The last objective is often secondary, on the one hand, because of the priority and emergency of the lesion process (dissection, metastases, and tumors of the base of the skull), and on the other hand, because of the discomfort with swallowing and phonation caused by paralysis that finally is little disabling, even in the absence of recovery [9] as in our two patients. In our two patients, the emergency lays in confirming carotid dissection and eliminating another, mostly cerebral impairment whose management differs from that of isolated paralysis of the half tongue.

The need for antithrombotic treatment to prevent a primary ischemic event or recurrence during arterial dissection is currently well established. This treatment includes anticoagulants or antiplatelets. At present, a superiority of one of the two therapies could not be demonstrated [12, 13]. A multicenter, randomized study, the cervical artery dissection in stroke study (CADISS), evaluated the efficacy of antiplatelet and anticoagulant therapies in extracranial spontaneous dissection [14]. The results of this study did not show any difference between the two treatments [14]. On the basis of this data, it is therefore lawful to use any of these agents in the presence of an extracranial dissection. Thus, our first patient benefited from platelet aggregating inhibitors treatment with acetylsalicylic acid unlike our second patient receiving low molecular weighted heparin anticoagulant treatment relayed by antivitamin K. Many centers offer routine aspirin in all cervical arterial dissections in view of the benefit and risk ratio including less side effects mainly less hemorrhagy risk, and it does not require biological follow-up unlike the antivitamin K. The duration of treatment depends primarily on the results of imaging performed during follow-up [15].

The prognosis of dissections of the internal carotid artery is generally good with a favorable evolution in more than 90% of cases [16]. Toko Djuidje et al., in France, in their observation on carotid dissection and isolated paralysis of the half tongue in a patient, noted the disappearance of symptoms including dysarthria and paresis of the tongue [2]. In both of our patients, the progression is also marked by the regression of the swallowing problem and paresis of the half tongue, four weeks after the introduction of platelet aggregating inhibitor treatment in our first patient and four weeks after the treatment of anticoagulant in our second patient.

## 5. Conclusion

Carotid dissection may be responsible for the damage to the lower cranial nerves. This is a very rare situation found in 8% of cases. The most commonly found damage to the hypoglossal nerve is observed in 5% of patients. When isolated, it can be unnoticed and delay the diagnosis of carotid dissection. Dissections of the cervical arteries and especially the carotid arteries are an important cause of stroke in young and middle-aged people accounting for 25% of strokes in this age group.

MRI, an main exam, must be performed as a matter of emergency in the face of diagnostic suspicion to confirm carotid dissection with a wall hematoma and its consequences on arterial flow in order to begin treatment.

The prognosis of dissections of the internal carotid artery is generally good with a favorable evolution in more than 90% of cases. In the face of any isolated paralysis of the half tongue, however, carotid dissection should be urgently sought and treated to reduce the risk of a transient or constituted ischemic accident.

## Data Availability

The data used to support the findings of this study are available within the article.

## Abbreviations

CADISS: Cervical artery dissection in stroke study
ICA: Internal carotid artery
INR: International normalized ratio
MRI: Magnetic resonance imaging.
